# Meyerson nevus triggered by COVID-19^⋆^

**DOI:** 10.1016/j.abd.2022.11.002

**Published:** 2023-04-27

**Authors:** Rubén Linares-Navarro, Pedro Sánchez-Sambucety, Manuel Ángel Rodríguez-Prieto

**Affiliations:** Department of Dermatology, University Hospital of León, León, Spain

Dear Editor,

Meyerson's nevus is an eczematous reaction on a melanocytic nevus. This reaction can also affect other types of lesions such as congenital nevi, dysplastic nevi, melanoma, and non-melanocytic lesions, in which case it is called Meyerson's phenomenon. It may be restricted to a single melanocytic nevus or affect several melanocytic nevi. In a small percentage of cases, it is accompanied by eczematous lesions in other locations. It usually resolves spontaneously or after treatment with topical corticosteroids. Its pathogenesis is not clear, and no triggers have been identified in most cases, although cases have been reported after sunburn or treatment with interferon-alpha alone or combined with ribavirin.^1^

Multiple cutaneous manifestations caused by COVID-19 have been described. However, its pathogenesis remains unclear. They have been attributed to an inflammatory mechanism, due to an immune response against viral nucleotides, and to a vascular mechanism, secondary to vasculitis or thrombosis.^2^ After a rigorous search of the literature, the authors did not find any case of Meyerson's nevus associated with COVID-19 infection or vaccination.

The authors present the case of a 34-year-old man with no medical history of interest who presented to the dermatology department with erythematous-squamous lesions and crusting over approximately half of his melanocytic nevi on the trunk (Fig. 1A). Visualization of dermoscopic structures was difficult due to the presence of serous crusting, slight whitish scaling, and an erythematous halo (Fig. 1B). The previous ten days he had suffered an uncomplicated upper respiratory tract infection due to COVID-19. The patient had not experienced similar lesions after SARS‐CoV‐2 vaccination or after previous infectious processes. Nor had he introduced any new drugs.

**Fig. 1:**
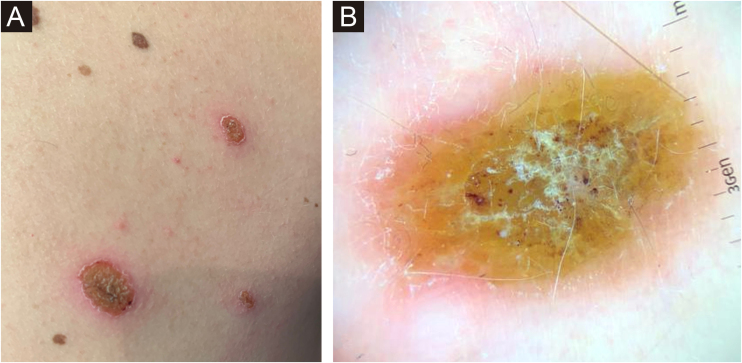
(A) Erythematous-squamous lesions and crusting over approximately half of his melanocytic nevi on the trunk. (B) Visualization of dermoscopic structures was difficult due to the presence of serous crusting, slight whitish scaling, and an erythematous halo

A biopsy of one of the lesions revealed nevus cells arranged in small thecae with adequate maturation without signs of dysplasia, surrounded by lymphocyte-predominant inflammatory infiltrate (55% CD4+, 45% CD8+) and marked spongiosis (Fig. 2).

**Fig. 2:**
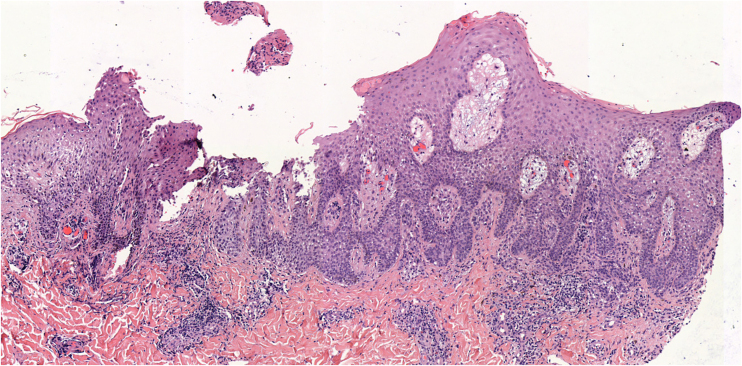
Biopsy of one of the lesions revealed nevus cells arranged in small thecae with adequate maturation without signs of dysplasia, surrounded by lymphocyte-predominant inflammatory infiltrate and marked spongiosis (Hematoxylin & eosin, ×50)

It was diagnosed as Meyerson nevus triggered by COVID-19 infection. The lesions resolved after topical corticosteroid treatment (methylprednisolone acetate once daily for two weeks) and have not recurred to date. The affected nevi show a typical pigmented reticular pattern at present.

As for nevocentric dermatoses in relation to SARS-CoV-2, a case of nevocentric erythema multiforme three days after COVID-19 vaccination (Comirnaty™ ‒ BioNTech/Pfizer ‒ Mainz, Germany/New York, NY, USA) has recently been reported.^3^ This is a different entity than Meyerson's phenomenon, but the pathogenesis of both could involve an interaction between Intercellular Cell Adhesion Molecule 1 (ICAM-1) and CD4 T-cells. ICAM-1 expression would be stimulated by certain interferons.^4^ SARS-COV-2 has proved to induce a type I interferon response in a subgroup of patients with mild disease,^5^ as in the case of our patient. This mechanism has been associated with the development of different autoimmune processes triggered by viruses and could explain the development of a Meyerson nevus coinciding with COVID-19 infection.

In conclusion, COVID-19 infection, through a mechanism mediated by ICAM-1 and interferon, could trigger Meyerson's nevus. The authors encourage the reporting of this type of skin reaction in order to consider their inclusion among the possible cutaneous manifestations related to COVID-19.

## Financial support

None declared.

## Authors’ contributions

Rubén Linares-Navarro: Approval of the final version of the manuscript; critical literature review; data collection, analysis, and interpretation; effective participation in research orientation; intellectual participation in propaedeutic and/or therapeutic; management of studied cases; manuscript critical review; preparation and writing of the manuscript; study conception and planning.

Pedro Sánchez-Sambucety: Approval of the final version of the manuscript; critical literature review; data collection, analysis, and interpretation; effective participation in research orientation; intellectual participation in propaedeutic and/or therapeutic; management of studied cases; manuscript critical review; preparation and writing of the manuscript; study conception and planning.

Manuel Ángel Rodríguez-Prieto: Approval of the final version of the manuscript; critical literature review; data collection, analysis, and interpretation; effective participation in research orientation; intellectual participation in propaedeutic and/or therapeutic; management of studied cases; manuscript critical review; preparation and writing of the manuscript; study conception and planning.

## Conflicts of interest

None declared.
